# β-Giardin as an Immunomagnetic Enrichment Target for Multi-Host Detection of *Giardia duodenalis* Cysts

**DOI:** 10.3390/pathogens14090918

**Published:** 2025-09-11

**Authors:** Hongyu Wang, Heng Yang, Chaofan Li, Mengge Chen, Xiaocen Wang, Xu Zhang, Pengtao Gong, Nan Zhang, Xichen Zhang, Jianhua Li, Xin Li

**Affiliations:** State Key Laboratory for Diagnosis and Treatment of Severe Zoonotic Infectious Diseases, Key Laboratory for Zoonosis Research of the Ministry of Education, Institute of Zoonosis, College of Veterinary Medicine, Jilin University, Changchun 130062, China; hongyuw23@mails.jlu.edu.cn (H.W.); yang_heng123@126.com (H.Y.); lichaofanxx@163.com (C.L.); chenmg1017@163.com (M.C.); wangxiaocen2016@163.com (X.W.); zhangxu1311@163.com (X.Z.); gongpt@jlu.edu.cn (P.G.); n_zhang@jlu.edu.cn (N.Z.); xczhang@jlu.edu.cn (X.Z.)

**Keywords:** *Giardia duodenalis*, cyst outer wall, proteomics, immunomagnetic beads

## Abstract

Giardiasis is a globally prevalent waterborne zoonosis. Rapid enrichment and detection technologies for this disease are essential. Cyst outer wall proteins are ideal targets for the enrichment and detection of cysts in the environment, but there are few available targets with suboptimal effectiveness. In this study, *Giardia duodenalis* (*G. duodenalis*) cysts were purified, and outer wall proteins were biotinylated, followed by streptavidin magnetic bead purification and mass spectrometry. Sixty-three novel cyst wall proteins were identified, and their functions were annotated through Gene Ontology (GO) and KEGG analyses. The β-giardin and α-1 giardin were among the newly identified and predicted to be located on the outer wall of *G. duodenalis* cysts. For the characterization of these two targets, we applied sequence analysis, prokaryotic expression, preparation of polyclonal antibodies, and determination of subcellular localization. Finally, based on β-giardin immunomagnetic beads were prepared using the polyclonal antibodies and tested for their enrichment efficiency. Immunomagnetic beads targeting β-giardin achieved 65% cyst enrichment efficiency in fecal samples, comparable to conventional methods. Clinical evaluation across 163 multi-host fecal samples (ferrets, Siberian tigers, red-crowned cranes) demonstrated concordance with nested PCR, successfully enriching cysts from PCR-positive specimens. The immunomagnetic beads method targeting β-giardin demonstrated effective *G. duodenalis* cyst enrichment in multi-host fecal samples. These results provide a proteomic framework for the cyst wall proteins of *G. duodenalis*, expanding the detection targets for *G. duodenalis* cysts. It also establishes a theoretical foundation for subsequent research on the composition and function of *G. duodenalis* cysts.

## 1. Introduction

Giardiasis is a globally prevalent waterborne zoonotic parasitic disease. It is caused by hosts ingesting food and water containing *Giardia* cysts [1]. *Giardia duodenalis* (*G. duodenalis*) is one of the most harmful pathogens spread through water sources. Patients mainly exhibit symptoms such as watery diarrhea and fever, and immunocompromised patients may die, especially those with AIDS and children [2]. It is reported that, the overall infection rate of *G. duodenalis* among children was calculated to be 18.3% in the African region. When analyzing the study population based on specific subgroups, it was found that the infection rate was highest among certain groups. Iron-deficient children had an infection rate of 65.2% (ranging from 61.3% to 69.1%), disabled children had an infection rate of 30.4% (ranging from 18.3% to 42.4%), HIV-infected children had an infection rate of 25.7% (between 11.2% and 40.2%), and displaced children had a prevalence of 20.2% (from 16.5% to 23.9%) [3]. Metronidazole and albendazole are the main treatments for *G. duodenalis* infections. However, these drugs can cause serious side effects, and treatment failure with Metronidazole, possibly due to resistant strains of *G. duodenalis*, have been reported [4].

The life cycle of *G. duodenalis* includes two stages: trophozoite and cyst. Trophozoites primarily reside in the host’s duodenum, then differentiate into cysts in the cecum, which represent the infective stage of *G. duodenalis*, and cysts are eventually excreted with feces [5]. Currently, three *G. duodenalis* cyst outer wall proteins have been reported: Cyst Wall Protein (CWP) 1–3 [6]. CWP1 and CWP2 share similar domains, including an N-terminal signal peptide and five tandem leucine-rich repeat (LRR) sequences [7]. In contrast, CWP3 contains only four complete LRRs [8]. CWP1 and CWP2 are primarily localized on the membranes of encystation-specific vesicles (ESVs) and throughout the entire cyst wall, whereas CWP3 is mainly localized inside the ESVs and within the hydrophobic cyst wall layer [7,8]. In recent years, there have also been reports indicating that the multiprotein bridging factor 1 (MBF1) gene plays a crucial role in regulating the expression of CWPs [9]. Additionally, *G. duodenalis* cyst wall protein 1 is a lectin that binds to curled fibrils of the GalNAc homopolymer [10].

Fecal cyst microscopic examination is the gold standard for the diagnosis of Giardiasis, and it is also the most widely used diagnostic method [11]. The detection of *G. duodenalis* has been enhanced through the utilization of antigen detection assays, several of which have been commercialized. A commercial direct fluorescent antibody (DFA) test has demonstrated a high sensitivity in detecting *G. duodenalis* [12]. In addition to DFA, immunochromatographic (IC) assays and enzyme immunoassays (EIAs) are also available for the detection of *G. duodenalis* [13,14]. IC assays are optimally suited for laboratories with lower capacities for diagnostic complexity, whereas EIA-based tests are more appropriate for high-throughput screening in areas with high prevalence [15]. The immunological enrichment and diagnosis method is an important supplement to the etiological diagnosis method, which has the advantages of strong specificity, high sensitivity and good stability [16]. In recent years, the imaging flow cytometry technology has also been developed for clinical diagnostics, environmental monitoring, and other potential biosensing applications [17].

In this study, we systematically characterized the proteomic composition of the outer wall of *G. duodenalis* cysts through streptomycin–biotin affinity chromatography purification. The physiological functions of identified proteins were elucidated using Gene Ontology (GO) and KEGG pathway analyses. To establish novel detection biomarkers, we prioritized cyst wall proteins with high species specificity for prokaryotic expression and polyclonal antibody development. Subsequent investigations focus on determining the subcellular localization patterns of these candidate proteins during encystation. Furthermore, the diagnostic potential of these proteins be validated by constructing antibody-based immunomagnetic bead systems for cyst enrichment from fecal samples. This comprehensive exploration is expected to advance our understanding of *G. duodenalis* encystation mechanisms while providing critical insights for developing next-generation detection platforms.

## 2. Materials and Methods

### 2.1. Ethics Statement

All animal experiments have received approval for research ethics from the Animal Welfare and Research Ethics Committee of Jilin University (IACUC Permit Number: 20160612). Three-week-old Specific Pathogen-Free (SPF) Mongolian gerbils were a gift from Professor Yuanhua Qin of Dalian Medical University. Six-week-old BALB/c mice were purchase from Liaoning Changsheng biotechnology co., Ltd. (Changsheng, Benxi, China). The animals were maintained in feeding cages with sterile food, water, and a 12 h light/dark cycle.

### 2.2. G. duodenalis Isolation, Cultivation, Encystation and Cysts Preparation

*G. duodenalis* trophozoites (WB strain, ATCC30957; GS/M clone H7 strain, ATCC50581; all purchased from the American Type Culture Collection, Manassas, VA, USA) were cultivated to the logarithmic growth phase within an optimized TYI-S-33 medium. A prechill was placed in an ice bath for 20 min, and the trophozoites were harvested through a centrifugal process at 1000× *g* for a period of 10 min. *G. duodenalis* was counted using a hemocytometer in preparation for subsequent experimental procedures. *G. duodenalis* were encysted using method as described previously [18].

1 × 10^7^ *G. duodenalis* trophozoites were administered to 5–6-week-old Mongolian gerbils via gavage to establish infection. Fecal samples were collected from the gerbils starting on the third day post-infection. *G. duodenalis* cysts were purified from feces using previously reported methodologies [19]. Briefly, the collected feces were dissolved in distilled water. The suspension was first filtered through a 100-mesh sieve to collect the filtrate. The filtrate was then centrifuged at 900× *g* for 10 min to pellet the cysts. The pellet was resuspended in a 33% zinc sulfate solution (MACKLIN, Shanghai, China) and thoroughly mixed before centrifugation at 400× *g* for 10 min. The supernatant was collected and diluted with four volumes of distilled water before centrifugation at 900× *g* for 10 min. The resulting pellet was resuspended in an appropriate amount of water and further purified by treatment with a 0.85 M sucrose solution (Guangfu reagent, Tianjing, China) to remove contaminants. Finally, the cysts were disinfected with a 10% sodium hypochlorite solution (HuaDong Product, Tianjing, China), yielding relatively clean *G. duodenalis* cysts. A hemocytometer was used to count the cysts, which were then utilized for subsequent experimental procedures.

### 2.3. Biotinylation of G. duodenalis Cyst Outer Wall Proteins and Purification of Biotinylated Proteins

The separation and purification of trophozoite surface proteins were performed essentially as previously described [20]. *G. duodenalis* cysts were resuspended in PBS (Sangon Biotech, Shanghai, China) solution. The cysts were counted using a hemocytometer and adjusted to a concentration of 1 × 10^8^ cysts/mL in PBS, with the addition of 1 mM protease inhibitor phenylmethanesulfonyl fluoride (PMSF) (Beyotime, Shanghai, China). The surface proteins of the cysts were labeled with 2 mM NHS-biotin (MedChemExpress, Shanghai, China) and incubated at 4 °C on a rotator (Biosharp, Hefei, China) for 2 h. After incubation, the samples were centrifuged at 900× *g* for 10 min and washed 3 times with cold sterile PBS to remove unbound NHS-biotin, and the pellet was resuspended in 1 mL cold sterile PBS (pH 8.0).

Following the biotinylation of the cyst surface proteins, we adhered to the purification method for biotinylated proteins. Briefly, the NHS-biotin labeled cysts were lysed using ultrasonication (200 W, 3 min working time). Subsequently, 200 mg of streptavidin magnetic beads (MedChemExpress, Shanghai, China) were added to the sample and incubated at 37 °C on a rotator for 90 min. The streptavidin magnetic beads bound to the surface proteins were enriched using a magnetic rack (Tiangen, Beijing, China) and washed 3 times with cold PBS.

### 2.4. Western Blot

Separated and purified outer wall protein samples, non-outer wall protein samples, and total *G. duodenalis* proteins were prepared with radioimmunoprecipitation assay lysis buffer with 1 mM PMSF. The proteins (30 μg/well) were separated by SDS-PAGE (12%) electrophoresis and transferred to PVDF membranes (Millipore, Bedford, MA, USA). Membranes were blocked with 5% skim milk for 2 h and incubated overnight at 4 °C with the following primary antibodies: rabbit anti-histone 3 (anti-H3) (Cell Signaling Technology, Danvers, MA, USA) rabbit anti-CWP1 (mouse polyclonal antibodies). After washing 3 times with PBS-0.05% Tween 20 (Biofroxx, Shanghai, China) for 10 min, the blots were incubated with secondary HRP-conjugated antibodies (Proteintech, Wuhan, China) for 1 h and washed as before. Finally, target proteins were visualized using ECL luminous reagent (Millipore, Bedford, MA, USA).

### 2.5. LC-MS/MS Analysis of the G. duodenalis Cyst Outer Wall Proteins

LC–MS/MS analysis was performed as previously described. Briefly, tryptic peptides were dissolved in 0.1% formic acid and analyzed using a Q Exactive™ Plus mass spectrometer (Thermo, Waltham, MA, USA) coupled to a UPLC system. The peptides were ionized by nano-electrospray ionization (NSI) and subjected to tandem mass spectrometry (MS/MS). The resulting tandem mass spectra were processed using the MaxQuant search engine (v.1.5.2.8). The database used for peptide sequence matching was UniProt *G. duodenalis* concatenated with a reverse decoy database. Cysteine alkylation was set as a fixed modification, while variable modifications included methionine oxidation, deamidation, and N-terminal acetylation. The false discovery rate (FDR) for protein and peptide-spectrum match (PSM) identification was set to 1%.

### 2.6. Bioinformatic Analysis

This study conducted a detailed analysis of the characteristics of the identified cyst outer wall proteins in *G. duodenalis* to comprehensively elucidate their functions. Differential proteins were screened using RStudio vision 4.4.1 (2024-06-14 ucrt). Functional annotations and GO analysis of these differential proteins were performed using the GiardiaDB website (https://giardiadb.org/giardiadb/) accessed on 13 June 2023, and the results were exported as tables. Finally, the results were visualized using the Microbiome Informatics website (http://www.bioinformatics.com.cn/) accessed on 13 June 2023. Additionally, KEGG analysis of the differential proteins was conducted using the GiardiaDB website, with the results exported as tables and visualized using the Microbiome Informatics website.

### 2.7. Production of Recombinant Proteins and Preparation of Polyclonal Antibodies

Total *G. duodenalis* trophozoites RNA was extracted using TRIZol (Tiangen, Beijing, China), and the cDNA was synthesized by using the TransScript One-Step RT-PCR SuperMix (Transgen Biotech, Beijing, China). The cDNA was used as the template to amplify the CWP1, α-1 giardin and β-giardin gene by PCR. The specific primers for CWP1, α-1 giardin and β-giardin amplification were shown in Table 1. PCR products of α-1 giardin and β-giardin were purified using a PCR purification kit (Sangon Biotech, Shanghai, China). The purified PCR products of the α-1 giardin and β-giardin gene sequence was inserted into the pGEX-4T-1 vector. The recombinant α-1 giardin and β-giardin protein was expressed in *Escherichia coli* (*E. coli*) BL21 (DE3) (Tiangen, Beijing, China) and confirmed by SDS-PAGE and Western blot. The recombinant pGEX-4T-1-α-1 giardin and pGEX-4T-1-β-giardin protein was purified by a GST-Tagged Protein Purification Kit (CoWin Biosciences, Beijing, China) according to the manufacturer’s instructions. The purified protein was emulsified with Freund’s complete adjuvant (Sigma Aldrich, St. Louis, MO, USA) and used to immunize six-week-old BALB/c mice by subcutaneous injection at the first time, and Freund’s incomplete adjuvant (Sigma Aldrich, St. Louis, MO, USA) was used at second and third times. After 3 immunizations, the blood was collected and stored at 4 °C overnight, and the serum was collected by centrifuging for 900× *g* for 10 min and stored at −20 °C.

### 2.8. Indirect Immunofluorescence Localization

Cover slides were placed into 24-well plates (Labselect, Hefei, China), and *G. duodenalis*-infected fecal dilution of gerbils was seeded. *G. duodenalis* cysts on cover slides permeated with 3% poly-L-lysine (Sigma Aldrich, St. Louis, MO, USA) were fixed with paraformaldehyde and then permeated with 0.25% Triton X-100 (Biofroxx, Shanghai, China) for 10 min. After blocking with 3% BSA (Biosharp, Hefei, China) for 2 h at room temperature, coverslips were then incubated with the anti-α-1 giardin polyclonal antibody (diluted in 3% BSA, prepared in mouse, 1:200), anti-β-giardin polyclonal antibody (diluted in 3% BSA, prepared in mouse, 1:200) and anti-CWP1 polyclonal antibody (diluted in 3% BSA, prepared in mouse, 1:200) at 4 °C overnight. After washing 3 times, the coverslips were incubated with Alexa Fluor 488 Dye conjugated anti-mouse secondary antibody (Proteintech, Rosemont, IL, USA, 1:200) for 1 h at room temperature. After the nucleis were counter-stained with DAPI (Servicebio, Wuhan, China), images were captured with a laser scanning confocal microscope (Fv1000; Olympus, Tokyo, Japan).

### 2.9. Preparation of Immunomagnetic Beads

Initially, 4 μg of the previously prepared β-giardin and CWP1 polyclonal antibody was biotinylated by co-incubation with 2 mM NHS-biotin (MedChemExpress, Shanghai, China). The biotinylated polyclonal antibody was then conjugated to streptavidin magnetic beads (MedChemExpress, Shanghai, China) following the manufacturer’s protocol. Briefly, 100 μL of magnetic beads were washed once with 1 mL of PBS-0.05% Tween 20 (Biofroxx, Shanghai, China) washing buffer. Subsequently, the beads were gently mixed and incubated with the biotinylated anti-β-giardin and anti-CWP1 polyclonal antibody at room temperature for 2 h with continuous agitation. After incubation, the anti-β-giardin and anti-CWP1conjugated magnetic beads were concentrated and separated from the background sample by placing the tube on a magnetic rack (Tiangen, Beijing, China). To remove unbound antibodies, the conjugated beads were washed twice with PBS-0.05% Tween 20 buffer using the same magnetic separation technique. Finally, the anti-β-giardin and anti-CWP1conjugated magnetic beads were resuspended in PBS and stored at 4 °C for further use.

### 2.10. Assessment of Immunomagnetic Beads Enrichment Efficiency

To evaluate the enrichment efficiency of the immunomagnetic beads, fecal samples were collected from gerbils experimentally infected with *G. duodenalis*. The gerbils were infected orally with *G. duodenalis* trophozoites and monitored for cyst shedding. Fresh fecal samples were collected during the peak shedding period, homogenized in PBS, and filtered through a 100-μm mesh to remove large debris. The filtrate was centrifuged at 900× *g* for 10 min, and the pellet was resuspended in PBS. The cyst concentration in the suspension was quantified using a hemocytometer.

The immunomagnetic bead enrichment procedure was performed as follows: 100 μL of magnetic beads were added to 500 μL of the fecal suspension containing *G. duodenalis* cysts. The mixture was gently rotated at room temperature for 2 h to allow the beads binding to the cysts. After incubation, the tube was placed on a magnetic rack (Tiangen, Beijing, China) for 5 min to separate the bead-bound cysts from the suspension. The supernatant was carefully removed, and the bead-cyst complex was washed twice with 1 mL of PBS-0.05% Tween 20 buffer to eliminate non-specifically bound debris. Finally, the bead-cyst complex was resuspended in 100 μL of PBS for further analysis.

To quantify the enrichment efficiency, the number of cysts in the initial fecal suspension and the final enriched sample was counted using a hemocytometer. Two operators count independently. If the difference between the groups exceeds 15%, a third party will conduct a re-count. The enrichment efficiency was calculated using the following formula:

Enrichment efficiency (%) = number of cysts in enriched sample/number of cysts in initial sample × 100%

### 2.11. Clinical Sample Collection

Fresh fecal samples were collected from three animal species: ferrets, Siberian tiger, and red-crowned cranes. Sampling was conducted in their natural habitats across multiple regions to ensure a representative collection. A total of 163 fecal samples were obtained, comprising 56 samples from ferrets, 75 from Siberian tiger, and 32 from red-crowned cranes. Samples were collected using sterile tools, placed in individually labeled sterile containers, and immediately transported to the laboratory on ice to preserve sample integrity.

### 2.12. Nested PCR

Genomic DNA was extracted from 200 mg of fecal specimens using the fecal genomic DNA extraction kit (Tiangen, Beijing, China) following the manufacturer’s protocol. Two-step nested PCR targeting the *G. duodenalis* β-giardin gene (Accession: GL50803_004812) was performed with primers. Primary PCR primers: F1: 5′-AAGCCCGACGACCTCACCCGCAGTGC-3′, R1: 5′-GAGGCCGCCCTGGATCTTCGAGACGAC-3′. Secondary PCR Nested primers: F2: 5′-GAACGAACGAGATCGAGGTCCG-3′, R2: 5′-CTCGACGAGCTTCGTGTT-3′.

The reaction mixture was 30 μL, including 10 × PCR buffer (TaKaRa, Beijing, China), 30 pM of each forward and reverse primer, 200 μM dNTPs (TaKaRa, Beijing, China), and 1 U Taq DNA polymerase (TaKaRa, Beijing, China), which was made up to 30 μL by nuclease-free water (TaKaRa, Beijing, China). The thermal cycling procedure consisted of 94 °C for 5 min, followed by 35 cycles of 95 °C for 30 s, 60 °C for 30 s, 72 °C for 30 s, and a final extension at 72 °C for 5 min. The amplification products were stained with ethidium bromide on a 1.0% agarose gel and visualized under UV light, and the 510 bp band was regarded as a positive sample.

### 2.13. Statistical Analysis

Experimental data were expressed as the mean ± SD of three independent experiments. One way analysis of variance (ANOVA) and *T*-test were used to evaluate the various detection conditions by using GraphPad Prism 6.01 (GraphPad Software, Inc., San Diego, CA, USA). Significance was set at * *p* < 0.05, ** *p* < 0.01, *** *p* < 0.001 and **** *p* < 0.0001.

## 3. Results

### 3.1. Proteomic Analysis of G. duodenalis Cyst Surface Protein

**Figure 1:**
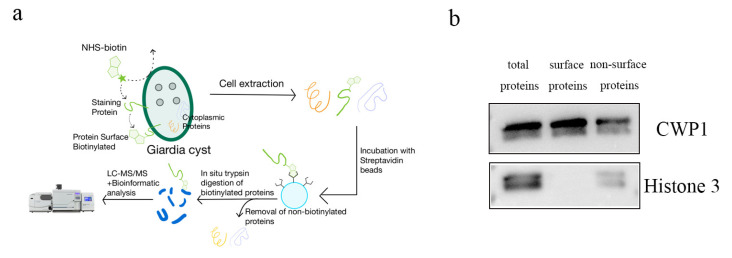
Biotin labeling of surface proteins in *G. duodenalis* cysts and verification. (**a**). Schematic representation of the purification of the outer wall proteins of *G. duodenalis* cysts. (**b**). Western blot validation of the outer wall proteins of *G. duodenalis* cysts.

The separation and purification method established for *G. duodenalis* trophozoite surface proteins was adapted to isolate and purify the cyst surface wall proteins for subsequent proteomic analysis. Collecting the feces of gerbils that were infected by *G. duodenalis* trophozoite. NHS-biotin was labeled on the surface of the cysts, followed by lysis of the organisms. Finally, streptavidin magnetic beads were used to purify the biotinylated outer wall proteins. The purification process is illustrated in the diagram (Figure 1a).

The purified outer wall proteins were validated by Western blot. The results show that the total proteins of *G. duodenalis* cysts and non-outer wall proteins of *G. duodenalis* cysts can detect the presence of the nuclear marker H3, while the purified outer wall proteins do not detect the presence of the nuclear marker H3. All samples can detect the presence of the identified outer wall protein CWP1 (Figure 1b). It indicates that the purification has yielded the outer wall proteins of *G. duodenalis* cysts. A total of 63 proteins were detected in the *G. duodenalis* cyst outer wall through mass spectrometry. The top identified 15 proteins are listed in Table 2.

**Table 1:** The primers of CWP1, β-giardin, α-1 giardin amplification for protein expression.

Gene ID	Name	Sequences (5′–3′)	Size (bp)	Application
GL50803_005638	CWP1-F	GGATCCATGATGCTCGCTCTCCTTGC	726	protein expression
	CWP1-R	GAATTCTCAAGGCGGGGTGAGGC		
GL50803_0011654	α-1 giardin-F	GGATCCATGCCGAAGGTCACCGAC	888	protein expression
	α-1 giardin-R	GAATTCCTACTTCACGCGCCAGAGG		
GL50803_004812	β-giardin-F	GGATCCATGTCTATGTTCACCTCCACCCG	819	protein expression
	β-giardin-R	GAATTCTTAGTGCTTTGTGACCATCGAGAGG		

**Table 2:** List of the identified top 15 proteins in this study.

Gene Name	Description	Previously Identified (Yes/No)	Prot Score
GL50581_1724	CWP2	Yes	128
GL50581_403	CWP1	Yes	101
GL50581_2806	CWP3	Yes	36
GL50803_00101168	Ankyrin repeat protein 1	No	124
GL50803_101291	Tubulin beta chain	No	114
GL50581_2741	Beta-giardin	No	77
GL50803_9515	Coiled-coil protein	No	75
GL50803_88765	Cytosolic HSP70	No	68
GL50803_15276	Glucosamine-6-phosphate isomerase	No	66
GL50803_0017043	Glyceraldehyde-3-phosphate dehydrogenase	No	59
GL50803 0011684	Leucine-rich repeat protein	No	49
GL50803_0017230	Gamma giardin	No	47
GL50803_0011654	Alpha-1 giardin	No	37
GL50803_13561	Protein Translation Elongation Factor 1B	No	47
GL50803_0013220	ATP-dependent RNA helicase	No	49

### 3.2. Functional Annotation of Proteins

**Figure 2:**
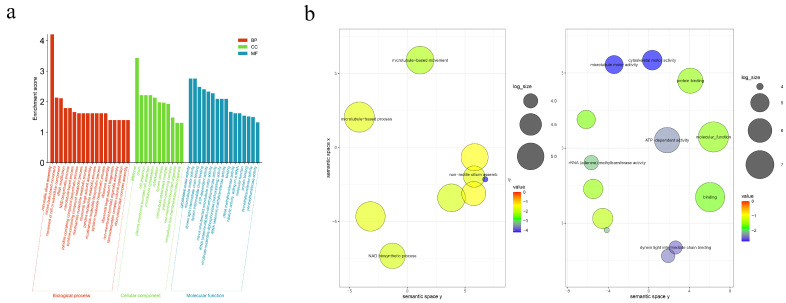
GO and KEGG pathway distribution of identified proteins in *G. duodenalis* cysts surface. (**a**) GO analyses of identified proteins under “biological process”, “cellular components”, “molecular function” in *G. duodenalis* cyst outer walls. (**b**) KEGG pathway distribution of identified proteins in *G. duodenalis* cyst outer walls.

To further elucidate the functions of these proteins, we conducted a GO analysis on the identified proteins with the UniProt-GOA data (http://www.ebi.ac.uk/GOA/) accessed on 13 June 2023. GO analysis of the obtained data predicted that the proteins are primarily involved in biological processes such as non-motile cilium assembly, microtubule base movement, and movement of cell or subcellular components. They are mainly localized in BBsome, cellular projection and plasma membranes. The biological functions include cytoskeletal motor activity, microtubule motor movement, and dynein light intermediate chain binding, among others (Figure 2a).

KEGG pathway enrichment analysis revealed that among the 63 identified proteins, 11 pathways were implicated. Among these, seven pathways were significantly enriched, including microtubule-based movement, NAD biosynthetic process, dynein light intermediate chain binding, cytoskeletal motor activity, microtubule motor activity, protein binding, ATP-dependent activity, and molecular function (Figure 2b).

### 3.3. Expression of Several Recombinant Surface Proteins

**Figure 3:**
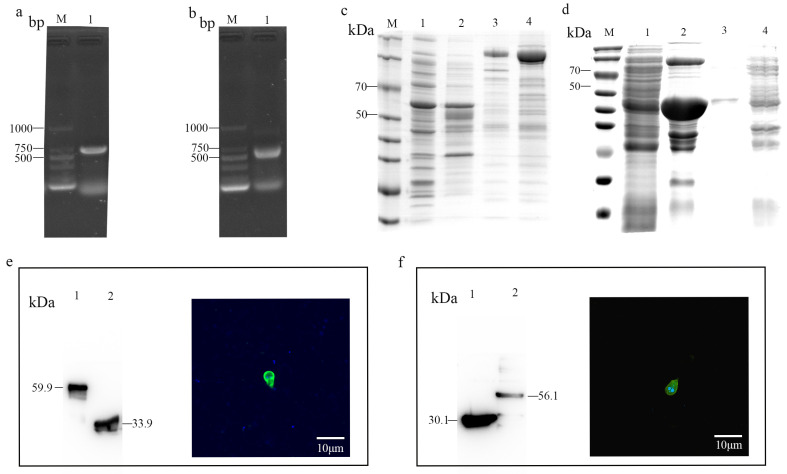
Expression of CWP1, α-1 giardin, β-giardin in *E. coli* and verification of polyclonal antibodies. (**a**). PCR amplification of α-1 giardin gene of *G. duodenalis*. Lane M: 1000-bp DNA ladder marker. Lane 1: α-1 giardin gene product. (**b**). PCR amplification of β-giardin gene of *G. duodenalis*. Lane M: 1000-bp DNA ladder marker. Lane 1: β-giardin gene product. (**c**). Analysis of expressed in *E. coli* revealed by SDS-PAGE. Lane 1: lysate supernatant from bacterial cells transformed with pGEX-4t-1-α-1 giardin with Isopropyl β-D-1-thiogalactopyranoside (IPTG) induction. Lane 2: lysate sediment from bacterial cells transformed with pGEX-4t-1-α-1 giardin with IPTG induction. Lane 3: lysate precipitation from bacterial cells transformed with pGEX-4t-1-α-1 giardin without IPTG induction. Lane 4: lysate from bacterial cells transformed with pGEX-4t-1. (**d**). Analysis of expressed in *E. coli* revealed by SDS-PAGE. Lane 1: lysate supernatant from bacterial cells transformed with pGEX-4t-1-β-giardin with IPTG induction. Lane 2: lysate sediment from bacterial cells transformed with pGEX-4t-1-β-giardin with IPTG induction. Lane 3: lysate precipitation from bacterial cells transformed with pGEX-4t-1-β-giardin without IPTG induction. Lane 4: lysate from bacterial cells transformed with pGEX-4t-1. (**e**). Western blot and immunofluorescence verification of α-1 giardin polyclonal antibodies. Lane 1: purified α-1 giardin recombinant protein. Lane 2: total protein of *G. duodenalis* trophozoites. (**f**). Western blot and immunofluorescence verification of β-giardin polyclonal antibodies. Lane 1: total protein of *G. duodenalis* trophozoites. Lane 2: purified β-giardin recombinant protein.

To gain further insight into the functionality of these proteins, β-giardin and α-1 giardin were selected for expression and polyclonal antibody preparation. Genomic DNA extracted from *G. duodenalis* WB strain was used for amplification of the genes which were subsequently cloned into the pGEX-4T-1 vector (Figure 3a,b). The recombinant proteins were expressed in *E. coli* BL21 (DE3) cells. SDS-PAGE analysis confirmed that the recombinant protein was expressed at their expected sizes: β-giardin (30.1 kDa), α-1 giardin (33.9 kDa) (Figure 3c,d).

Polyclonal antibodies were prepared by immunizing BALB/c mice with a mixture of the respective protein and Freund adjuvant. After validation through Western blot, it was observed that the antibody effectively bound to the protein displaying correct molecular weights (Figure 3e,f). Notably, the apparent molecular weights of the recombinant proteins were significantly higher than those detected in the *G. duodenalis* total extract. This size difference of approximately 26 kDa is consistent with the presence of the N-terminal glutathione S-transferase (GST) tag (molecular weight ~26 kDa) fused to the recombinant proteins during expression and purification. This confirms that the antibodies specifically recognize both the recombinant GST-tagged proteins and their native counterparts in *G. duodenalis* lysates. Meanwhile, immunofluorescence was used to test the specific binding of the prepared polyclonal antibodies to *G. duodenalis*. The results showed that both β-giardin and α-1 giardin could bind to the surface of the trophozoites (Figure 3e,f).

### 3.4. β-Giardin and α-1 Giardin Are Located in G. duodenalis Cyst Surface

**Figure 4:**
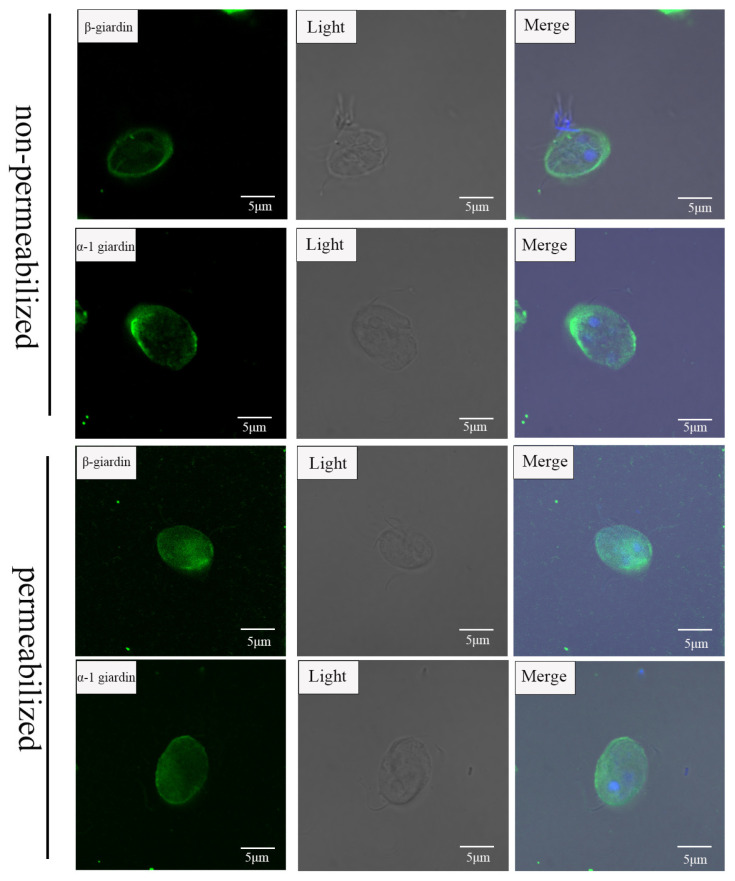
Immunofluorescence microscopy detection of α-1 giardin, β-giardin in *G. duodenalis* cyst stage using the mouse polyclonal antibodies. Immunofluorescence microscopy detection of β-giardin and α-1 giardin in *G. duodenalis* cyst stage using the mouse anti-β-giardin and α-1 giardin antibody.

The subcellular localization of giardins in *G. duodenalis* cysts was determined using anti-giardin antibodies and laser confocal microscopy. Both permeabilized and non-permeabilized *G. duodenalis* cysts were labeled with anti-giardin antibodies. It was observed that both β-giardin and α-1 giardin could be labeled on the outer surface of the cysts that had not been permeabilized (Figure 4). This indicates that the newly identified two giardins are located on the surface of the cysts.

### 3.5. Detectability of Selected Conserved Surface Proteins

**Figure 5:**
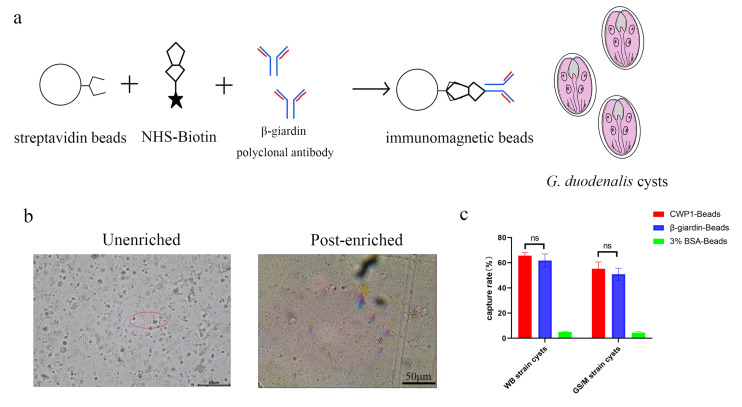
Preparation of immunomagnetic beads for *G. duodenalis* and optimization of enrichment conditions. (**a**). Schematic of the preparation and enrichment of *G. duodenalis* β-giardin immunomagnetic beads. (**b**). *G. duodenalis* cysts enriched by immunomagnetic beads. (**c**). Comparison of the enrichment efficiency of anti-β-giardin and anti-CWP1 immunomagnetic beads for different Giardia strains. The data are presented as the mean ± SEM from at least three independent experiments. ns, not significant.

Given that β-giardin and α-1 giardin are positioned on the surface of the outer wall of *G. duodenalis* cysts, this indicates that these giardins have the potential to serve as new detection targets for *G. duodenalis*. This study selected β-giardin as a target for immunomagnetic bead preparation, based on its species specificity and localization (Figure 5a). *G. duodenalis* cysts can be enriched by immunomagnetic beads, and eluted using acidic conditions (Figure 5b). The optimization of enrichment conditions for immunomagnetic beads was systematically investigated by evaluating enrichment efficiency under different antibody dilution ratios and incubation durations. The results demonstrated that the highest enrichment efficiency was achieved at an antibody dilution ratio of 1:200 with an enrichment duration of 60 min (Table 3 and Table 4). The maximum enrichment efficiency of immunomagnetic beads reached 65% under optimized conditions (antibody dilution ratio at 1:200, 60 min incubation) (Table 5). Based on its species specificity and localization, β-giardin was selected as the detection target for immunomagnetic bead preparation. Subsequently, the enrichment efficiency of immunomagnetic beads coated with anti-β-giardin antibodies was compared to those functionalized with anti-CWP1 antibodies for both WB and GS/M strains under optimal conditions. The results show that the prepared immunomagnetic beads can effectively enrich different strains. There was no significant difference in the enrichment efficiency of anti-β-giardin and anti-CWP1 immunomagnetic beads (*p* > 0.05) (Figure 5c). These findings highlight the critical influence of temporal and concentration parameters on the performance of immunomagnetic separation systems.

### 3.6. Clinical Performance Evaluation of Giardia Cysts via Immunomagnetic Beads Detection

**Figure 6:**
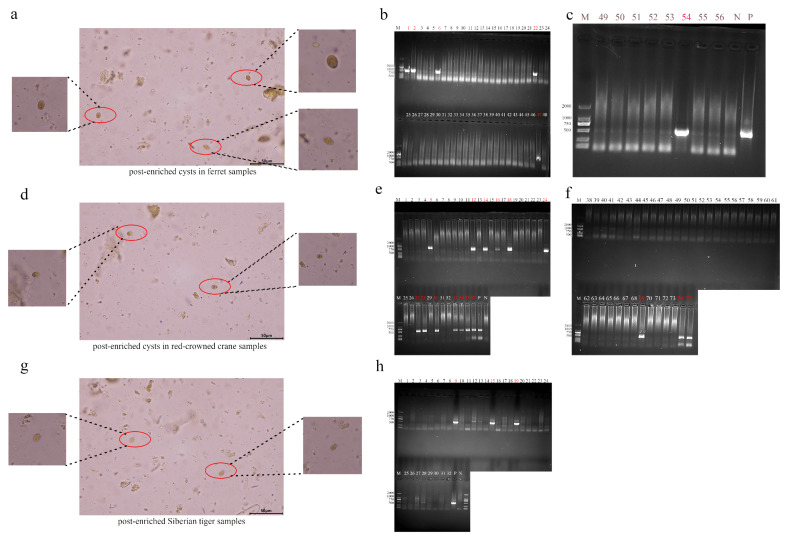
Nested PCR assay and cysts enrichment of clinical samples for Giardia (**a**). Cysts in ferret fecal samples. (**b**,**c**). Nested PCR assay of ferret fecal samples. (**d**). Cysts in Siberian tiger fecal samples. (**e**,**f**). Nested PCR assay of Siberian tiger fecal samples. (**g**). Cysts in red-crowned crane fecal samples. (**h**). Nested PCR assay of red-crowned crane fecal samples. M: 2000-bp DNA ladder marker. Lane 1- XX: PCR products of fecal genomic BG genes from different ferret, Siberian tiger and red-crowned crane. *p*: Positive control (PCR amplification of BG gene of *G. duodenalis*). N: Negative control (PCR amplification of BG gene of water).

To evaluate the clinical efficacy of the developed immunomagnetic beads detection method for *Giardia* cysts, the method was applied to detect cysts in fecal samples from ferrets, Siberian tigers, and red-crowned cranes. A total of 163 fresh clinical fecal specimens were collected, comprising 56 ferret samples, 75 Siberian tiger samples, and 32 red-crowned crane samples. All specimens underwent nested PCR amplification targeting the β-giardin locus. The positivity rate was 10.7% (6/56) (Figure 6b,c) in ferret samples, 21.3% (16/75) (Figure 6e,f) in Siberian tiger samples, and 9.3% (3/32) (Figure 6h) in red-crowned crane samples. Subsequent immunomagnetic bead enrichment of positive samples successfully detected *Giardia* cysts in all PCR-positive specimens (Figure 6a,d,g). Concurrently, the enrichment efficiency was evaluated by processing cysts spiked into 5-g fecal samples under optimized conditions, followed by cyst counting in the enriched samples (Table 6).

The results demonstrate that the immunomagnetic beads prepared in this study can effectively enrich *G. duodenalis* cysts in feces. These giardins have the potential to become new detection targets for novel etiological detection methods.

## 4. Discussion

*G. duodenalis* is a globally prevalent zoonotic protozoan parasite responsible for causing diarrhea [21]. The cyst stage represents its primary infectious phase, and the cyst wall plays a crucial role as a protective barrier, enabling the parasite to endure adverse environmental conditions while maintaining its viability and infectivity [22]. Despite its biological significance, the specific protein composition of the cyst wall has remained largely uncharacterized. This study led to the identification of 63 novel cyst wall proteins in *G. duodenalis* and elucidated the localization of β-giardin and α-1 giardin on the cyst surface. Furthermore, immunomagnetic beads were successfully developed using polyclonal antibodies against β-giardin, achieving an enrichment efficiency of 65% for *G. duodenalis* cysts in fecal samples.

The cyst wall of *G. duodenalis* is a complex structure composed of approximately 40% proteins, with the remainder consisting of carbohydrates, such as N-acetylgalactosamine, and lipids [23]. It is well established that the cyst wall primarily contains three major cyst wall proteins (CWPs): CWP1, CWP2, and CWP3, which are exclusively localized on the cyst surface [6]. In addition to these CWPs, other proteins have also been identified within the cyst wall. For instance, High Cysteine Non-Variant Cyst Protein (HCNCp) is a recently discovered cyst protein that localizes not only to the cyst wall but also to the cyst interior [24]. By using *Cryptosporidium parvum* oocyst wall protein sequences as a reference, an Epidermal Growth Factor-like Cyst Protein (EGFCP) was identified in the *G. duodenalis* genome database (Chiu et al., 2010) [25]. These proteins are all highly expressed during the encystation process, highlighting their potential roles in cyst formation and stability. In the current study, the cyst wall proteomics analysis identified CWP1, CWP2, and CWP3, which are key constituents of the cyst wall. However, HCNCp and EGFCP were not detected in the analysis. This discrepancy may be attributed to strain differences or the purity of the cyst wall protein preparations used in the study. The identification of the cyst wall proteome presented here provides novel insights into the composition, structure, and function of the *G. duodenalis* cyst wall, and it lays the foundation for further investigation into the mechanisms underlying cyst formation and environmental resilience.

The currently reported *G. duodenalis* cyst wall proteins, including CWP1-3, EGFCP1, and HCNCp, are characterized by the presence of signal peptides and a high cysteine content [7,24]. However, sequence analysis of the two giardins (β-giardin and α-1 giardin) did not reveal the presence of signal peptides. This suggests that these proteins may be transported to the cyst wall surface via encystation-specific vesicles (ESVs) during the encystation process, similar to the transport pathway of CWP1. CWP1 and CWP2 are primarily distributed on the membranes of ESVs and throughout the cyst wall, while CWP3 is mainly localized within ESVs and the water-resistant layer of the cyst wall [7,8]. HCNCp is predominantly found in the nucleus and co-localizes with CWPs in ESVs and the cyst wall during encystation [24]. Similarly, EGFCP1 is localized in ESVs, the cyst wall, and the cyst interior, indicating a potential shared transport pathway among these proteins [25]. This study demonstrated that, without permeabilization, both β-giardin and α-1 giardin localize to the outer surface of the cyst wall. After permeabilization, they are also distributed within the cytoplasm. The functions of these giardins have been shown to involve maintaining the cytoskeleton. However, their detailed roles in cyst wall formation and stability remain to be elucidated. Further studies using overexpression or gene knockout approaches are needed to clarify the specific mechanisms by which these proteins contribute to the structural integrity and functionality of the cyst wall.

The detection of CWP1 in the “non-surface protein” fraction is indeed attributable to the inherent limitations of NHS-biotin labeling. While NHS-biotin effectively labels surface-exposed lysine residues, its efficiency depends on protein accessibility, steric hindrance, and local chemical environments. CWP1, despite being a major cyst wall component, may contain partially buried domains or exhibit uneven biotinylation due to structural heterogeneity. This could result in incomplete labeling of all surface-localized CWP1 molecules, allowing unlabeled portions to persist in the “non-surface” fraction after streptavidin pulldown.

The US Environmental Protection Agency’s “Method 1623” is the standard protocol for detecting *G. duodenalis* cysts in water samples using immunomagnetic beads [26]. However, compared to immunomagnetic beads used for bacterial or viral enrichment, which can achieve over 70% efficiency, the enrichment efficiency for *G. duodenalis* cysts typically ranges from 30% to 50% [16,27,28,29]. This lower efficiency is likely due to the larger size of *G. duodenalis* cysts, which often require multiple beads to bind effectively to the cyst surface for successful capture. The low-concentration loss is mainly due to the complex environment in the feces, where the cysts and residual debris form a co-precipitate that is difficult to separate, and it becomes even more difficult to separate at low concentrations. Advancements in nanomaterials offer promising opportunities to develop new magnetic bead materials that could enhance pathogen binding and improve overall detection efficiency [30]. Additionally, identifying more cyst wall protein targets in *G. duodenalis* could provide additional binding sites for immunomagnetic beads, thereby increasing the likelihood of capturing cysts.

Current immunomagnetic separation (IMS) techniques for pathogen detection still face several limitations. The enriched samples still require microscopic examination or other auxiliary detection methods. To address these constraints, numerous integrated approaches combining IMS with other detection techniques (e.g., ELISA, LAMP) have been developed, which enhance detection specificity and field applicability [31,32]. Furthermore, IMS demonstrates suboptimal performance when processing samples with low target pathogen abundance. Perhaps it is due to the insufficient sample size, making it difficult to conduct the test using a microscope. Notably, recent studies have reported that coupling IMS with biosensors significantly reduces detection time [33]. Additionally, the capture efficiency of IMS varies across different parasite strains. In the study, polyclonal antibodies generated against the WB strain genome showed superior enrichment efficiency for WB strain cysts compared to GS/M strain cysts, likely due to variations in protein epitopes among different strains. Therefore, when selecting target antigens, both species specificity and degree of conservation across different strains should be carefully considered.

Moreover, combining immunomagnetic beads with other detection methods could significantly enhance the identification of *G. duodenalis* cysts in samples. For example, immunomagnetic beads (IMB)-enzyme-linked immunosorbent assay (ELISA) can first concentrate the pathogens and then provide quantitative detection of pathogens [34]. Similarly, coupling immunomagnetic beads with nucleic acid amplification techniques such as Loop-mediated Isothermal Amplification (LAMP) and Recombinase Polymerase Amplification (RPA) could enable more sensitive and rapid detection of *G. duodenalis* cysts [35,36]. These integrated approaches leverage the strengths of multiple techniques to overcome the limitations of individual methods, offering a more robust and reliable means of detecting low-concentration pathogens in complex environmental samples. Future research should focus on optimizing these combined methods and exploring novel materials to further improve the detection efficiency and sensitivity for *G. duodenalis* cysts in water.

## 5. Conclusions

This study identified 63 novel cyst wall proteins of *G. duodenalis*, providing a proteomic framework for cyst wall composition and function. β-giardin and α-1 giardin were localized on cyst surfaces and used to develop immunomagnetic beads with 65% enrichment efficiency in fecal samples. These findings expand detection targets for *G. duodenalis* cysts and lay the foundation for improved diagnostic tools and further research.

## Figures and Tables

**Table 3:** Evaluation of IMB efficiency in cysts capture with different concentration of antibody.

Sample	Antibody Dilution Radio	Estimated Number of Cysts Captured by IMB	IMB Efficiency	Optical Microscopy Observation
S1	1:100	1.0 × 10^4^	41%	+
S2	1:200	1.25 × 10^4^	52%	+
S3	1:500	5 × 10^3^	20%	+
S4	1:1000	5.0 × 10^3^	20%	+
S5	1:2000	2.5 × 10^3^	10%	+

Estimated by microscopy observation. Initial number of cysts in 500 μL is 2.4 × 10^4^. Incubation time between IMB and cysts is 60 min. “+” indicates that the cysts can be observed under the microscope, but precise counting is not possible.

**Table 4:** Evaluation of IMB efficiency in cysts capture with different capture time.

Sample	Incubation Time Between IMB and Cysts	Estimated Number of Cysts Captured by IMB	IMB Efficiency	Optical Microscopy Observation
S1	10 min			+
S2	30 min	7.5 × 10^3^	31%	+
S3	60 min	1.25 × 10^4^	52%	+
S4	120 min	1.0 × 10^4^	41%	+
S5	180 min	7.5 × 10^3^	31%	+

Estimated by microscopy observation. Initial number of cysts in 500 μL is 2.4 × 10^4^. Antibody dilution ratio is 1:200. “+” indicates that the cysts can be observed under the microscope, but precise counting is not possible.

**Table 5:** Evaluation of IMB efficiency in cysts capture.

Sample	Estimated Number of Cysts in 500 μL	Estimated Number of Cysts Captured by IMB	IMB Efficiency	Optical Microscopy Observation
S1	1 × 10^4^			+
S2	2 × 10^4^	1.75 × 10^3^	8.5%	+
S3	5 × 10^4^	1.25 × 10^4^	25%	+
S4	1 × 10^5^	6.5 × 10^4^	65%	+
S5	2 × 10^5^	1.05 × 10^5^	52.5%	+

Estimated by microscopy observation. Antibody dilution ratio is 1:200. Incubation time between IMB and cysts is 60 min. “+” indicates that the cysts can be observed under the microscope, but precise counting is not possible.

**Table 6:** Valuation of IMB efficiency in clinical samples.

Sample	Unriched Cysts in 1 mL	Post-Enriched Cysts Captured by IMB	IMB Efficiency
Ferret	1.25 × 10^4^	6.25 × 10^3^	50%
Red-crowned crane	8 × 10^3^	3.5 × 10^3^	43.75%
Siberian tiger	1.5 × 10^4^	8.25 × 10^4^	55%

Estimated by microscopy observation.

## Data Availability

The datasets generated during the current study are available from the corresponding author on reasonable request.
